# An augmented reality overlay for navigated prostatectomy using fiducial-free 2D–3D registration

**DOI:** 10.1007/s11548-025-03374-5

**Published:** 2025-05-08

**Authors:** Johannes Bender, Jeremy Kwe, Benedikt Hoeh, Katharina Boehm, Ivan Platzek, Angelika Borkowetz, Stefanie Speidel, Micha Pfeiffer

**Affiliations:** 1 Department of Translational Surgical Oncology, NCT/UCC Dresden, Dresden, Germany; 2 German Cancer Research Center (DKFZ), Heidelberg, Germany; 3 Faculty of Medicine and University Hospital Carl Gustav Carus, TUD Dresden University of Technology, Dresden, Germany; 4 Helmholtz-Zentrum Dresden-Rossendorf (HZDR), Dresden, Germany; 5 Department of Urology, University Hospital Carl Gustav Carus, TU Dresden, Dresden, Germany; 6 Department of Radiology, University Hospital Carl Gustav Carus, TU Dresden, Dresden, Germany; 7 National Center for Tumor Diseases (NCT/UCC Dresden), Dresden, Germany; 8 University Medical Centre Rostock, University of Rostock, Rostock, Germany

**Keywords:** Navigation system, Prostatectomy, Augmented reality, 2D–3D registration

## Abstract

**Purpose:**

Markerless navigation in minimally invasive surgery is still an unsolved challenge. Many proposed navigation systems for minimally invasive surgeries rely on stereoscopic images, while in clinical practice oftentimes monocular endoscopes are used. Combined with the lack of automatic video-based navigation systems for prostatectomies, this paper explores methods to tackle both research gaps at the same time for robot-assisted prostatectomies.

**Methods:**

In order to realize a semi-automatic augmented reality overlay for navigated prostatectomy, the camera pose w.r.t. the prostate needs to be estimated. We developed a method where visual cues are drawn on top of the organ after an initial manual alignment, simultaneously creating matching landmarks on the 2D and 3D data. Starting from this key frame, the cues are then tracked in the endoscopic video. Both PnPRansac and differentiable rendering are then explored to perform 2D–3D registration for each frame.

**Results:**

We performed experiments on synthetic and in vivo data. On synthetic data differentiable rendering can achieve a median target registration error of 6.11 mm. Both PnPRansac and differentiable rendering are feasible methods for 2D–3D registration.

**Conclusion:**

We demonstrated a video-based markerless augmented reality overlay for navigated prostatectomy, using visual cues as an anchor.

**Supplementary Information:**

The online version contains supplementary material available at 10.1007/s11548-025-03374-5.

## Introduction

Radical prostatectomy comes with multiple risks, including incontinence, impotency and other operative complications. Although nerve-sparing surgery can partly mitigate them, it has a higher risk of worse oncological outcome. On the other hand, intraoperative visualization of sensitive structures such as tumours or neurovascular bundles could possibly improve both functional and oncological outcome. One intuitive solution to this task could be the use of augmented reality on a screen or directly inside the console of the surgery robot.

In order to make laparoscopic navigation systems a clinical reality, these systems need to be easy to set up and accurate, and should ideally work with commonly available hardware. This paper explores the field of urology, more specifically radical prostatectomy, as an exemplary application area to develop methods that aim for these goals. Most approaches in this field use other modalities besides the endoscopic video stream, oftentimes ultrasound [1–3], but also optical [4] or magnetic [5] tracking as well as fluorescence [6]. However, these modalities are disruptive as they are usually not part of the clinical workflow. Furthermore, the existing methods that focus mainly on video as input usually require continuous interaction with the surgeons as can be seen in the following.

Porpiglia et al. [7, 8] describe an approach which relies on manually moving, rotating and scaling a 3D model of the patient’s prostate throughout the surgery. The rendered image is then overlaid on the endoscopic video stream. In addition, the user can bend and stretch the virtual prostate to mimic the current deformation of the real prostate. In a similar way, in Ukimura et al. [9] a 3D model is manually oriented and displayed alongside the endoscopic video. Using a convolutional neural network, Tanzi et al. [10] segment the bladder catheter in a specific phase of the surgery. The obtained mask is then used to predict its anchor point and orientation to finally overlay the prostate model correctly. Building upon that, Padovan et al. [11] extends the usage of the CNN to the estimation of rotation of the catheter. Under ideal conditions, this works also without a visible catheter. This leads to the goal of exploring navigation systems that (a) rely only on video information and (b) minimize manual interaction by the users. In order to strive for a generally applicable solution, a monocular approach based on 2D–3D registration was chosen.

These registration methods have gained popularity for other surgical navigation tasks, such as liver surgery [12], neurosurgery [13] and surgery of the uterus [14]. These methods often rely on detection of landmarks in 3D and 2D modality [12, 14, 15], which can be very challenging in prostatectomy, as geometric features are rare and contours are difficult to identify uniquely. Other methods rely on texture or shading cues [13, 16], which may fail due to occlusion of the target structures.

## Methods

We propose a novel approach for a video-based markerless augmented reality overlay for navigated prostatectomy. It uses flexible user-defined cues on anatomical structures which are tracked in the endoscopic video stream and rigid 2D–3D registration based on differentiable rendering in order to estimate the pose of the anatomical structures with respect to the virtual camera. It is an easy-to-set up semi-automatic approach that requires some manual interaction in a key frame and no user input afterwards.

**Fig. 1:**
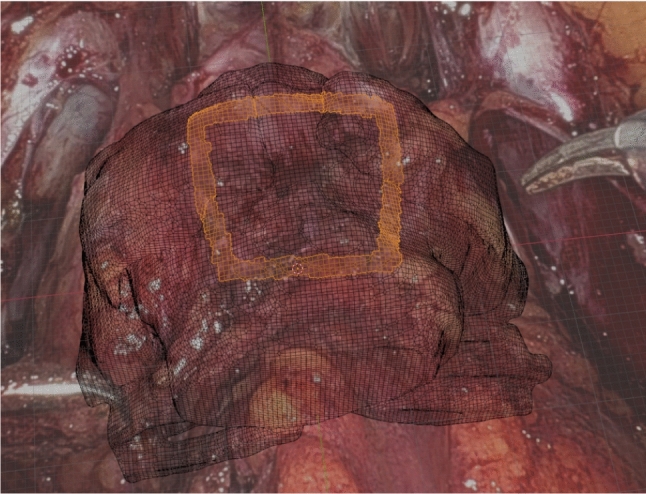
A cue is drawn on the surface of the prostate and neurovascular bundles. The selected faces are used to create a 3D mesh of the cue, and the projected image is used as a mask for tracking it throughout the surgery

### Initial steps

A 3D model is created from the preoperative MRI scan of the prostate and its neighbouring structures, the neurovascular bundles and vesicular glands. Afterwards, in a key frame chosen for initialization, two steps have to be completed in order to start the automatic tracking: first, the 3D model is manually aligned to match the visible anatomical structures in the endoscopic video stream. Afterwards, visual cues are drawn on top of (Fig. 1). This approach allows simultaneous acquisition of both 2D and 3D data about the cue. The obtained segmentation mask is used as an initialization to track the cue in subsequent frames, while a 3D mesh of the cue is constructed by projecting the 2D cue onto the preoperative model.

### Tracking

**Fig. 2:**
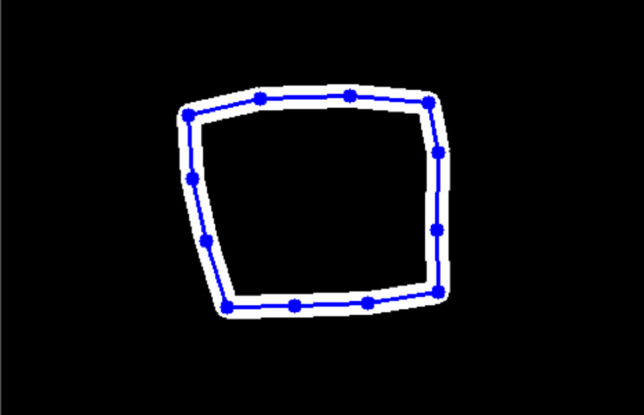
Masks of the cue are created by drawing lines between the tracked key points

The drawn cues are approximated by key points connected with lines as shown in Fig. 2. For tracking these points in the endoscopic video stream, CoTracker [17] is used. It is a method which is initialized with a set of coordinates of points and the corresponding image and predicts the position of the key points in the following frames.

### Registration

Two different methods for 2D–3D registration are investigated: PnPRansac and differentiable rendering. The former is used as a baseline.

Differentiable rendering [18] is a method where the standard forward pass of a rendering pipeline is modified in such a way that it is differentiable. The rendered output is then compared with a reference image to compute an image loss in 2D, which can back-propagated through the render pipeline to optimize the desired parameters.

For this paper, a differentiable renderer was implemented using PyTorch3D [18]. It draws only the silhouette of the given cue and then calculates the mean squared error between the rendered image and the 2D mask provided by the tracker described above. This loss is then minimized by optimizing the parameters position and orientation of the cue in the virtual camera coordinate space by using Adam [19]. More specifically, the orientation is expressed in a 6D representation by Zhou et al. [20]. The estimated pose with the lowest loss is chosen and used as an initialization for the next frame. This procedure is then repeated until the end of the video.

User-defined cues on the surface of the organ instead of silhouettes of anatomical structures were selected as features for the 2D–3D registration in order to minimize occlusion issues. For instance, rendering the silhouette of a 3D model of the isolated prostate could create a mask that includes regions that are always obscured by other organs such as the bladder in the intraoperative view. The resulting difference between rendered and reference mask is then challenging to handle in the differentiable rendering process.

### Experiments

**Fig. 3:**
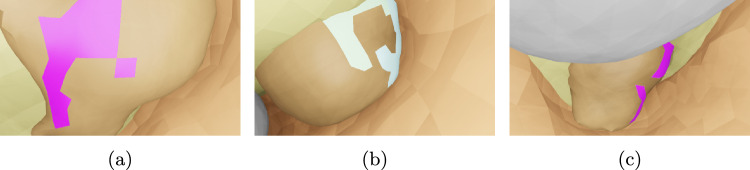
Examples of rendered images from synthetic dataset. The pink and white faces on the organ-like shape are the cues, fat is shown in light yellow, the abdominal wall is shown in brown and additional organ-like shapes are shown in grey

To validate the described method, experiments were carried out on two different datasets:

**Synthetic dataset:** The synthetic dataset comprises of 504 scenes of generated, random organ-like 3D shapes (simulating the intraoperative state of an organ) inside a larger, hollow shape (simulating the inflated abdominal cavity). The shapes have an average size of 26.1 mm. The cues are randomly generated on the surface of the organ. In each scene, images of the simulated organ and the cues are rendered from 15 different randomly placed camera poses. Examples of such images are shown in Fig. 3. The method was developed on a split of 4 scenes, in the following named as the parameterization set and a much larger test set of 500 scenes. To create a realistic scenario, both splits were filtered to discard samples with too small or too large cues and samples where cues are occluded by other anatomical structures. This leads to a total of 15 frames across all scenes in the parameterization set and 45 frames in the test set, respectively.

**In vivo dataset:** Videos of the staging phase of two robot-assisted prostatectomies with the DaVinci Xi (Intuitive Surgical, Sunnyvale, USA) were captured at the University Hospital Carl Gustav Carus in Dresden. To obtain the camera parameters, a calibration using a method from Hardner et al. [21] was performed before each surgery. In addition, for each patient, a preoperative MRI was carried out. Important anatomical structures like prostate, neurovascular bundles and vesicular glands were manually annotated by a radiologist in 3D Slicer [22] and a patient-specific 3D model built. Similar to the synthetic dataset, patient 1 was used for parameterization and patient 2 for evaluation. In addition, 5 salient key points were manually annotated in both patient videos every 1 s as a ground truth for evaluating the tracker.

**Fig. 4:**
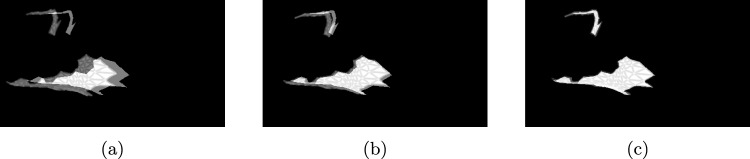
An example of an alignment of a cue rendered by the differentiable renderer (moving mask) to the reference obtained from the scene (stationary mask). Initial position: **a**, during alignment: **b**, final position: **c**

**Experiments on synthetic dataset:** We used the synthetic dataset to parameterize the differentiable rendering approach for camera alignment. Due to the synthetic nature of the data, perfect cues are available, allowing us to use the samples for parameterization without the effects of noise in any manually annotated labels.

To simulate the pose of the camera before alignment, noise was applied to the ground-truth pose. More specifically, uniformly sampled noise between −5 and 5 mm was added to each coordinate of the position of the camera. In addition, to each Euler angle of the rotation of the camera uniformly sampled noise between −5 and $5^\circ$ was added. For each frame, this procedure was repeated 3 times on the parameterization set and 5 times on the test set. On this dataset, we treat each camera pose individually as the camera does not follow a smooth path but is placed randomly for each frame.

To find suitable learning rates for translation and rotation, these hyperparameters were optimized on the parameterization set using Optuna [23]. The cost for this parameter search was defined as the lowest loss during the alignment of one frame averaged over all frames.

Afterwards, with the best parameters chosen according to this criterion, the method was evaluated on the test set. For each sample, the initial and aligned camera pose was compared with the ground truth with respect to the translation and rotation error. In addition, the target registration error was calculated for each organ-like shape based on 100 points randomly sampled inside the mesh.

**Experiments on in vivo dataset:** For each patient in the in vivo dataset, the described initial steps were carried out for the first frame, resulting in a manually aligned ground-truth camera pose and a corresponding mask and key points in 2D as well as the mesh of the cue and key points in 3D. This information was then used to apply both PnPRansac and differentiable rendering for all following frames in the recording. For the latter method, we use the alignment result of frame i as the initialization for frame i+1, as the camera performs a continuous movement. Learning rates were first optimized on patient 1 analogous to the approach on the synthetic dataset, then fixed and applied to patient 2. Furthermore, CoTracker was evaluated quantitatively by applying it not only to the annotated ground-truth key points in the first frame but also on additional constructed key points. These are derived by placing them on the midpoint on all connecting lines between the annotated ones in order to not skew the results by using key points that are easily trackable.

## Results

**Fig. 5:**
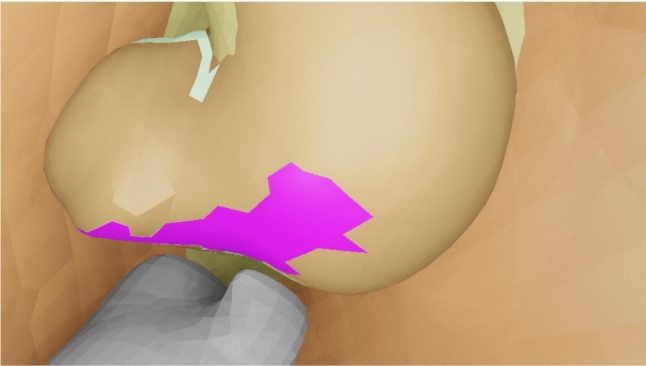
Rendering of the scene used for the alignment in Fig. 4. The reference mask corresponds to the silhouette of the coloured cues

**Fig. 6:**
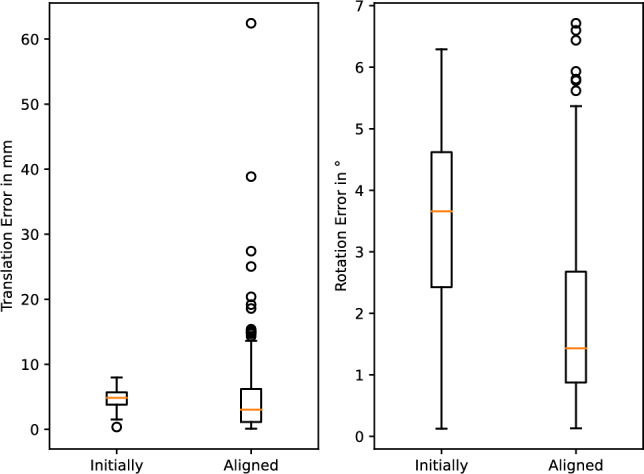
Application of differentiable rendering to the test set of the synthetic dataset: translation and rotation error of the initial and aligned camera pose each with respect to the ground-truth pose

The application of the differential rendering-based approach to a sample of the test set of the synthetic dataset is shown in Figs. 4 and 5, whereas the distribution of the metrics over all samples is shown in Fig. 6. The median translation error of the camera pose was reduced from 4.86 to 3.04 mm and the median rotation error from 3.66 to $1.43^\circ$. The median target registration error after alignment is 6.11 mm.

**Fig. 7:**
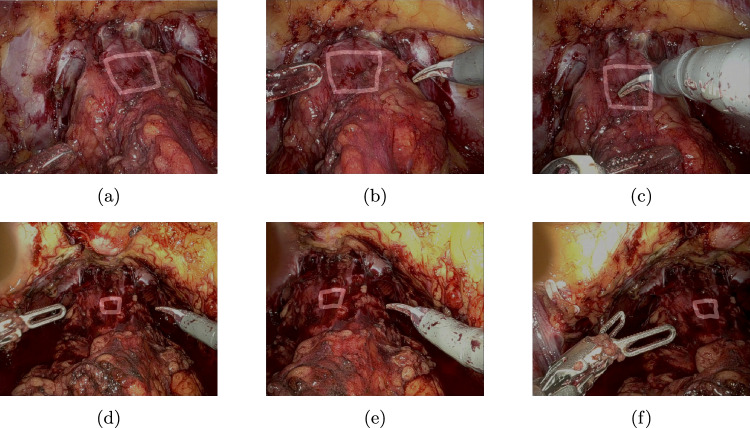
Results of tracking the rectangle cue throughout the video—top row **a**–**c**: patient 1, bottom row **d**–**f**: patient 2

**Fig. 8:**
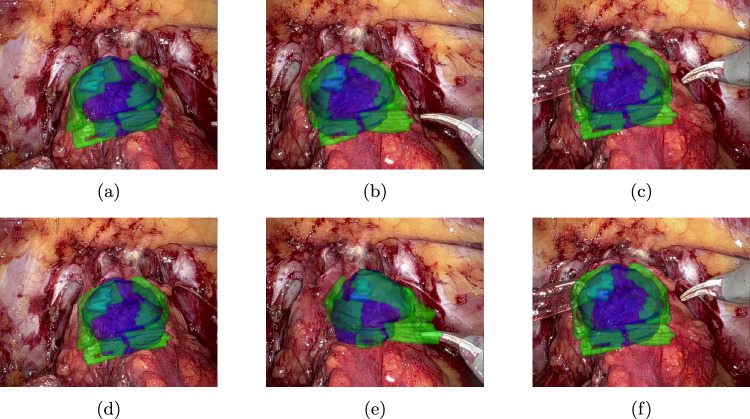
Augmented reality overlay for patient 1 (parameterization) using the rectangle cue—top row **a**–**c**: PnPRansac, bottom row **d**–**f**: differentiable rendering

**Fig. 9:**
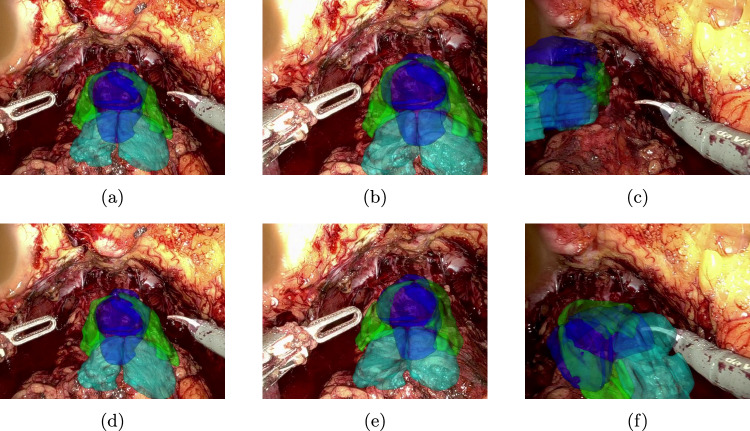
Augmented reality overlay for patient 2 (test) using the rectangle cue—top row **a**–**c**: PnPRansac, bottom row **d**–**f**: differentiable rendering

**Fig. 10:**
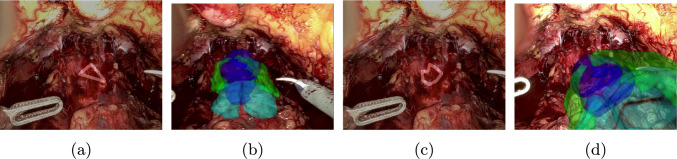
Augmented reality overlay for patient 2 (test) using the triangle **a**, **b** and complex shape cue **c**, **d**—both using differentiable rendering

The median tracking error of CoTracker regarding the aforementioned constructed key points is 2,46 px for patient 1 and 5,36 px for patient 2. The qualitative results of tracking a rectangle cue on in vivo data are shown in Fig. 7, whereas the corresponding augmented reality overlay based on the predicted camera pose of the 2D–3D registration is displayed in Figs. 8 and 9. In addition, results for a triangle and a more complex shape similar to the cues in the synthetic dataset are shown in Fig. 10. Frames were selected that show the behaviour of the methods on key moments of the camera trajectory.

## Discussion

Our proposed method depends on a good initial alignment, which is currently performed manually. We have an ongoing clinical trial in which we want to determine its feasibility. In the future, this step could be replaced by an automatic method such as Koo et al. [12].

The measured tracking errors as well as the qualitative results presented in Fig. 7 show that a CoTracker-based approach allows a robust and relatively accurate tracking of a cue that can handle fast camera movements, deformation as well as occlusion by the instruments. This provides a very good basis for the 2D–3D registration that builds upon it.

The results on the test set of the synthetic dataset show that an alignment method based on differentiable rendering using the silhouette of a cue is feasible. The median target registration error is in the acceptable range of 5–10 mm for tumours in navigated prostatectomy, showing potential for future clinical application. However, the accuracy of the alignment could be further improved. Two causes for the remaining error were identified: first, the image is blurred by the differentiable renderer so that a change in camera pose has a less noisy effect on the loss. This reduces however the accuracy as the rendered cue is systematically diluted on the edges. Here, a better trade-off between blur parameters and optimization behaviour could be found.

Second, as the synthetic dataset is designed to cover a general setup, edge cases can occur that were not identified before. The sample that has the highest translation error in Fig. 6 for example could not be aligned correctly as the virtual camera was not able to render the cue properly as it is too close to the cue after random perturbation.

On in vivo data, both PnPRansac and differentiable rendering are able to track the movement of the anatomical structures. Misalignments of the differentiable renderer for the rectangular cue can be see in Figs. 8e and 9f, where the predicted rotation overshoots or the estimated cue flips counterclockwise. The latter can be prevented by choosing a non-rotationally symmetric cue like the non-equilateral triangle (see Fig. 10b). The differentiable rendering approach is also less robust than PnPRansac if key points are not tracked properly anymore (see Fig. 10d). A failure case of the PnPRansac approach is shown in Fig. 9c. Here, the organ suddenly switches to a different solution of the pose estimation. Probable cause is the fact that if the 3D points lie in a plane, there exist two symmetrical solutions for the camera pose that lead to the same 2D projection. This effect seems to occur also if the condition is only partially met by the chosen cue. Both methods can still follow the anatomical structures during deformation as shown in Fig. 8c and f.

PnPRansac works naturally with key points, whereas differentiable rendering provides a flexible framework where different types of information gathered from the scene can be processed, such as masks, contours and depth. In addition, as the whole rendering pipeline is differentiable, other parameters could be optimized in the future as well, for example the position of the vertices themselves, opening up the possibility to capture deformation.

## Conclusion

A proof of concept for a video-based markerless augmented reality overlay for navigated prostatectomy was shown. Adding cues on the fly that are tracked throughout the surgery in combination of both PnPRansac and differentiable rendering provide a 2D–3D registration framework that does not rely on the visibility and correct segmentation of certain anatomical structures. However, due to its flexible nature, it could also be an option to use automatically detectable cues in addition to user-defined ones. The possibility of interaction could also allow the user to correct the predicted cues. Furthermore, the ability to optimize the vertices of the organ’s 3D model opens up the chance to capture its deformation.

## Supplementary information

Six videos are provided alongside the paper: the first three use the rectangle cue, while the last three use both triangle and complex shape cue: results of tracking for both patients (Online Resource (OR) 1, corresponding to Fig. 7), augmented reality overlay for patient 1 (OR 2, Fig. 8) and patient 2 (OR 3, Fig. 9), as well as results of tracking for patient 2 (OR 4, Fig. 10a, c) and augmented reality overlays for patient 2 for differentiable rendering (OR 5, Fig. 10b, d) and PnPRansac (OR 6).

## Supplementary Information

Below is the link to the electronic supplementary material.

Supplementary file 1 (mp4 38535 KB)

Supplementary file 2 (mp4 27714 KB)

Supplementary file 3 (mp4 47038 KB)

Supplementary file 4 (mp4 47623 KB)

Supplementary file 5 (mp4 49392 KB)

Supplementary file 6 (mp4 47524 KB)
